# Retrograde insertion of the outback reentry device from a tibial artery for complex infrainguinal recanalization

**DOI:** 10.1186/s42155-019-0088-7

**Published:** 2019-12-30

**Authors:** Lorenzo Patrone, Ondrej Stehno

**Affiliations:** 1 West London Vascular and Interventional Center, Northwick Park, St Mark’s Hospitals, Watford Road, HA1 3UJ, Harrow, Middlesex, London, UK; 2 Vascular Surgery Department, Na Homolce Hospital, Roentgenova 2/37, Prague 5, Czech Republic

**Keywords:** Outback, Infrainginal, Retrograde, Re-entry, Tibial vessels

## Abstract

**Introduction:**

Recanalization in an antegrade fashion in complex infrainguinal arterial disease can prove challenging and use of the Outback re-entry device via a retrograde approach may be an option.

**Case presentation:**

Using an antegrade approach we were unable to cross the native occluded superficial femoral artery in a patient after two occluded bypasses, with ulcers and was unfit for general anaesthesia.

We successfully attempted retrograde re-entry using an Outback device via a phantom segment of the anterior tibial artery. After angioplasty and stenting a satisfactory result was achieved with one artery runoff.

**Conclusions:**

At the 24 months follow up reconstruction was patent and ulcers were healed after 4 months.

The outcome of more cases will be decisive for the broader applicability of this technique.

## Introduction

Antegrade recanalization and distal re-entry is often challenging in complex infrainguinal disease, particularly in patients with previous surgery.

Use of a re-entry device is an option in these situations (Smith et al. 2011). One of the popular choices among re-entry devices is the Outback LTD Re-entry Catheter (Cordis, Miami Lakes, Florida). Despite the limitations of the Outback device (Shin et al. 2011) it is frequently used for infrainguinal re-entry (with reported success 65–100%) and is even used to re-enter true lumen of below-the-knee vessels (Diamantopoulos et al. 2018; Kitrou et al. 2015). The Outback device has also proved useful in chronic and even calcific lesions of the superficial femoral artery (Gandini et al. 2013). If it proves impossible to cross the lesion or make a re-entry in an antegrade fashion, a retrograde approach may be an option.

We present a case of retrograde recanalization of the anterior tibial artery, popliteal artery and superficial femoral artery with retrograde re-entry via the short superficial femoral artery stump using an Outback device inserted from a phantom segment of the anterior tibial artery.

## Case presentation

An 81 y-old patient presented with extensive infected leg ulcers and a rest pain. He had a history of one occluded femoropopliteal proximal bypass and two occluded femoropopliteal distal bypasses to the anterior tibial artery (ATA) and the tibioperoneal trunk (TPT) respectively.

The patient was referred to us for an attempt of endovascular recanalization under local anaesthesia. The patient, given his comorbidities, was unfit for general anaesthesia.

**Fig. 1:**
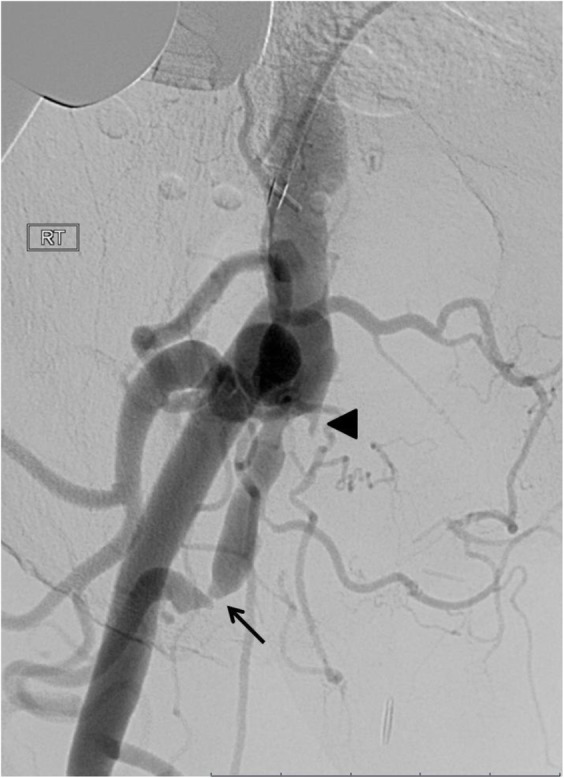
Baseline angiogram showing the native superficial femoral artery occluded few centimetres after the origin (arrow) with a big collateral coming off from the stump. A small stump of the previous femoro-popliteal bypass is also visible (arrow head)

The right leg angiogram showed occlusion of the superficial femoral artery (SFA) with a short stump and a large collateral arising at a 45 degree angle (Fig. 1). The Popliteal artery (POP), the TPT and the posterior tibial artery were also occluded. The ATA was recanalised by collateral just after its origin to occlude again few centimetres below. ATA and peroneal artery (recanalised by collaterals at the level of its mid segment) were the vessels reaching the foot (ATA occluded at the dorsalis pedis passage) with posterior tibial artery recanalised at the ankle level by collaterals.

The right common femoral artery (CFA) was punctured in an antegrade fashion but any attempt to cross the native occluded SFA failed, being unable to engage the true lumen of the occluded SFA or even to start a subintimal dissection, despite the use of different combinations of wires and catheters..

**Fig. 2:**
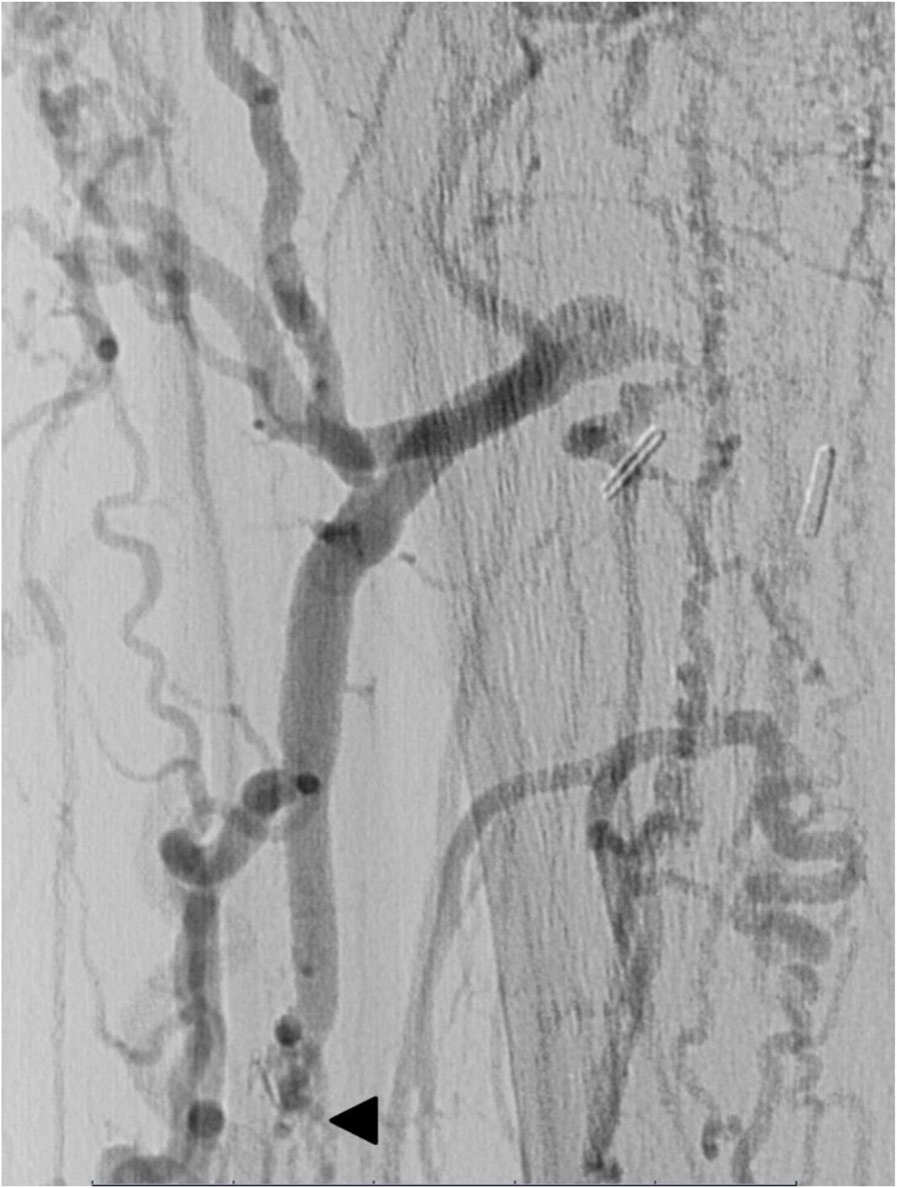
Magnified view at the level of the proximal anterior tibial artery which looks recanalised by collaterals coming from branches of the deep femoral artery and then occluded few centimetres after its origin (arrow head). No flow is seen in the popliteal artery

The blind segment of the ATA was then punctured in a retrograde fashion under fluoroscopic guidance by a 21G needle (Fig. 2).

A V18 wire (Boston Scientific, Natick, Massachusetts), supported just by an 0.018 CXI catheter (Cook, Bloomington, Indiana) managed to reach the proximal SFA stump subintimally but all attempts at re-entry into the true lumen at that level failed.

**Fig. 3:**
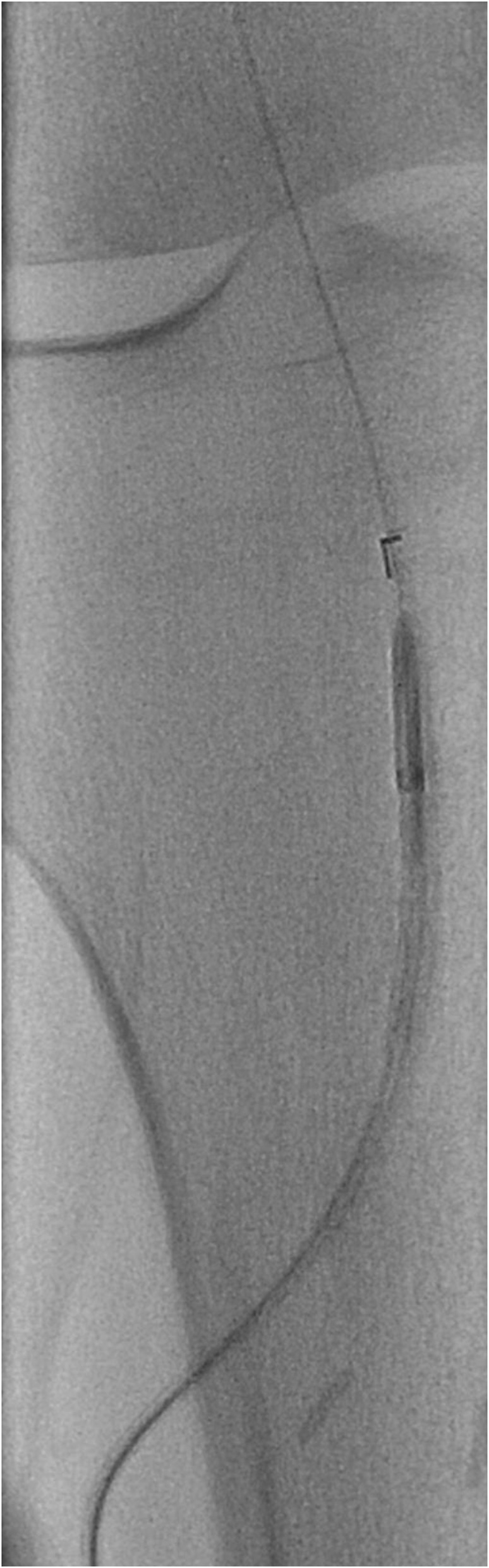
Outback catheter re-entry device inserted sheath less from the anterior tibial artery access over an 0.18 wire

**Fig. 4:**
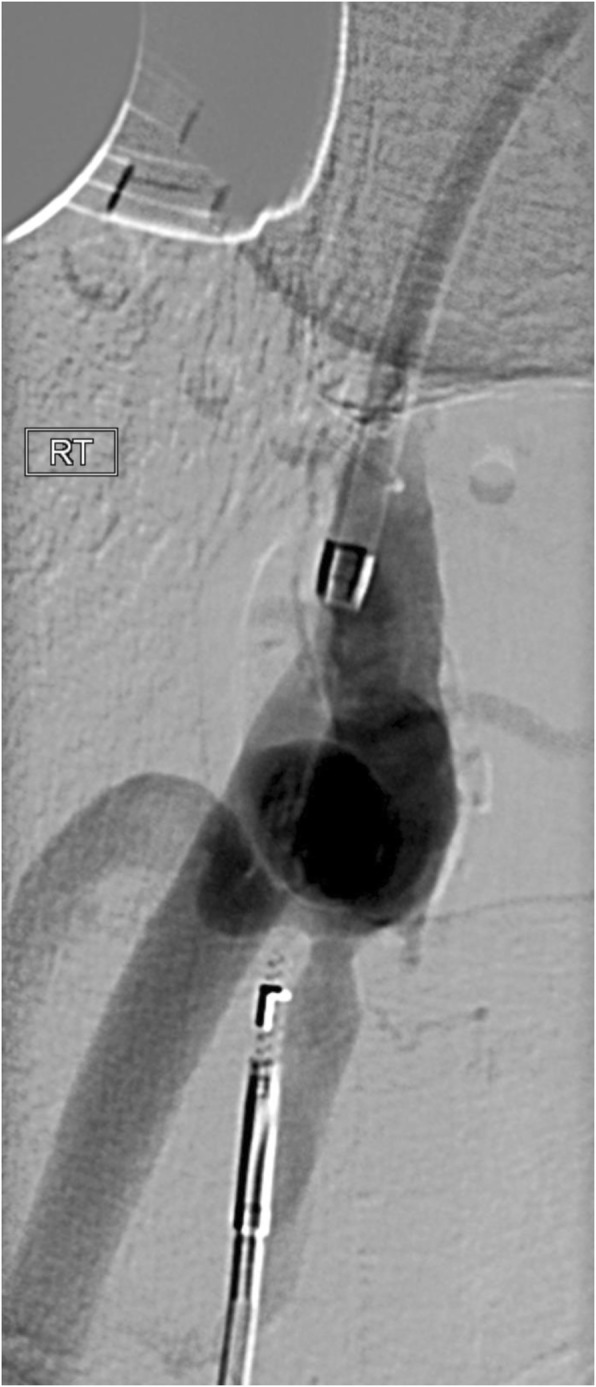
Outback catheter re-entry device positioned at the level of the superficial femoral artery stump before successful re-entry into that vessel

The Outback re-entry device was then passed sheath-less from the ATA access on the 0.018 wire (Fig. 3). The Outback was aimed at the proximal stump of the SFA (Fig. 4), the re-entry was successful on the first attempt and the wire was snared out from the CFA sheath.

The distal access was then removed and a 3 mm balloon was inflated at that level for 3 min to seal the vessel.

The short occluded segment of the ATA just below entry site was recanalised intraluminally from the CFA access and stented using a 3.5 mm XIENCE Prime BTK Drug Eluting Stent (Abbott, Chicago, Illinois).

After subintimal angioplasty of the SFA, POP and proximal ATA, the latter was stented by a 4 mm XIENCE Prime BTK Drug Eluting Stent (Abbott, Chicago, Illinois). The POP and SFA also required stenting, performed by overlapping 5.5 mm Supera Stents (Abbott, Chicago, Illinois) and 6 mm Absolute Pro stent (Abbott, Chicago, Illinois) at the level of the proximal SFA.

## Results

**Fig. 5:**
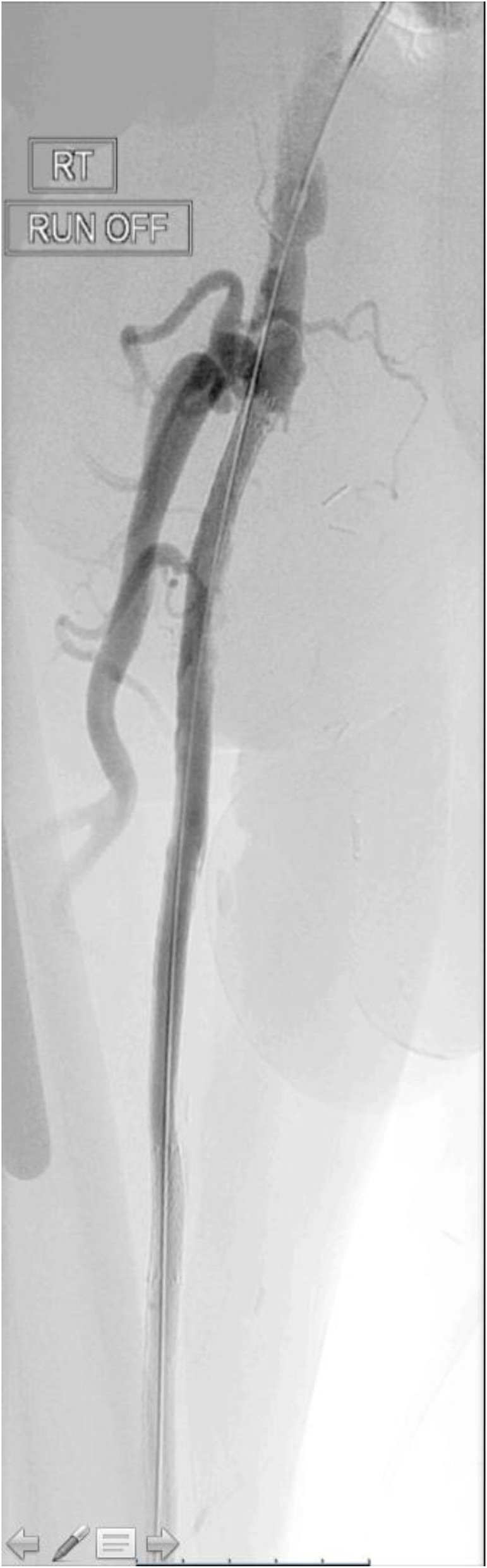
SFA runoff post procedure part 1

**Fig. 6:**
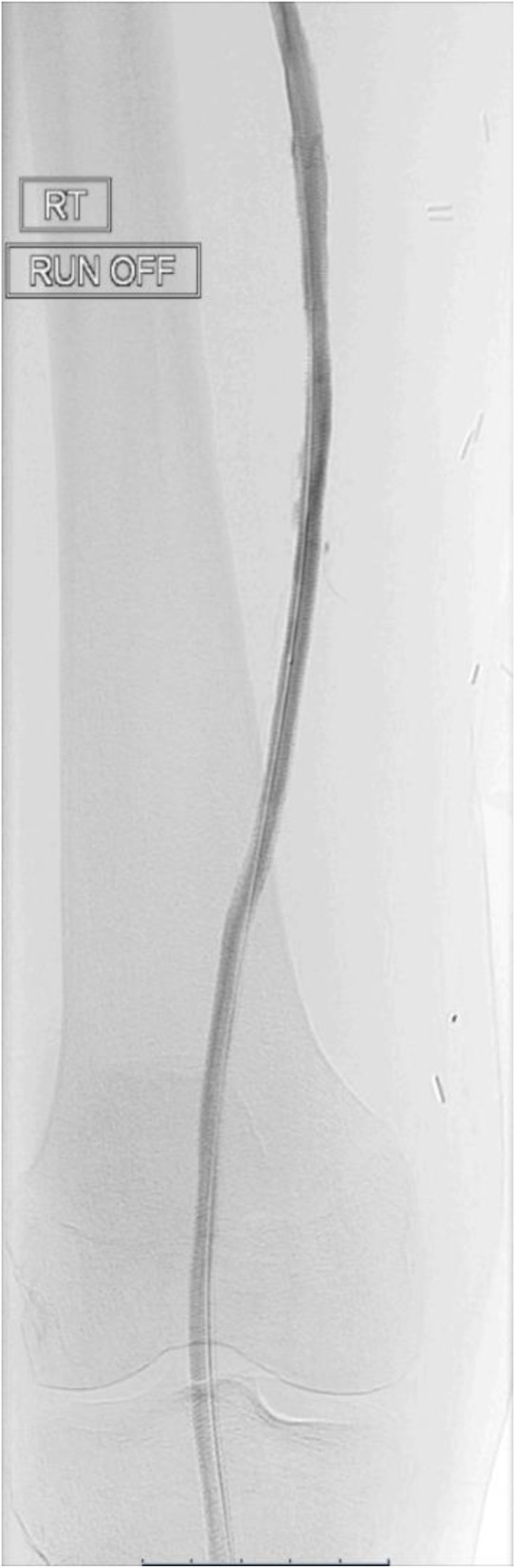
SFA runoff post procedure part 2

**Fig. 7:**
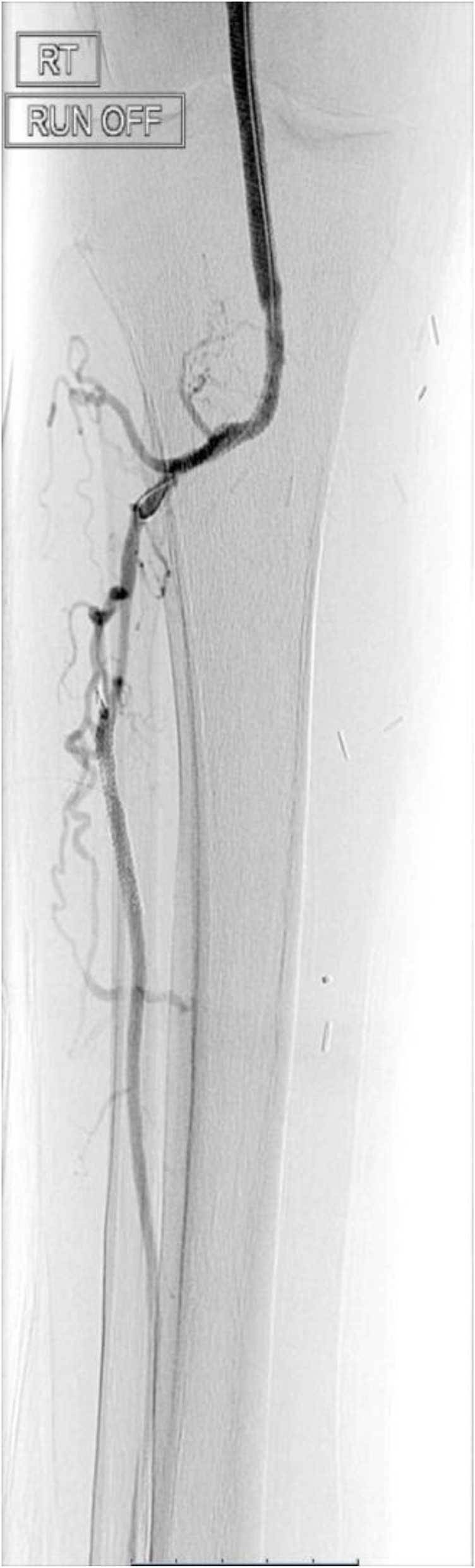
Final runoff of the distal popliteal artery and the anterior tibial artery

**Fig. 8:**
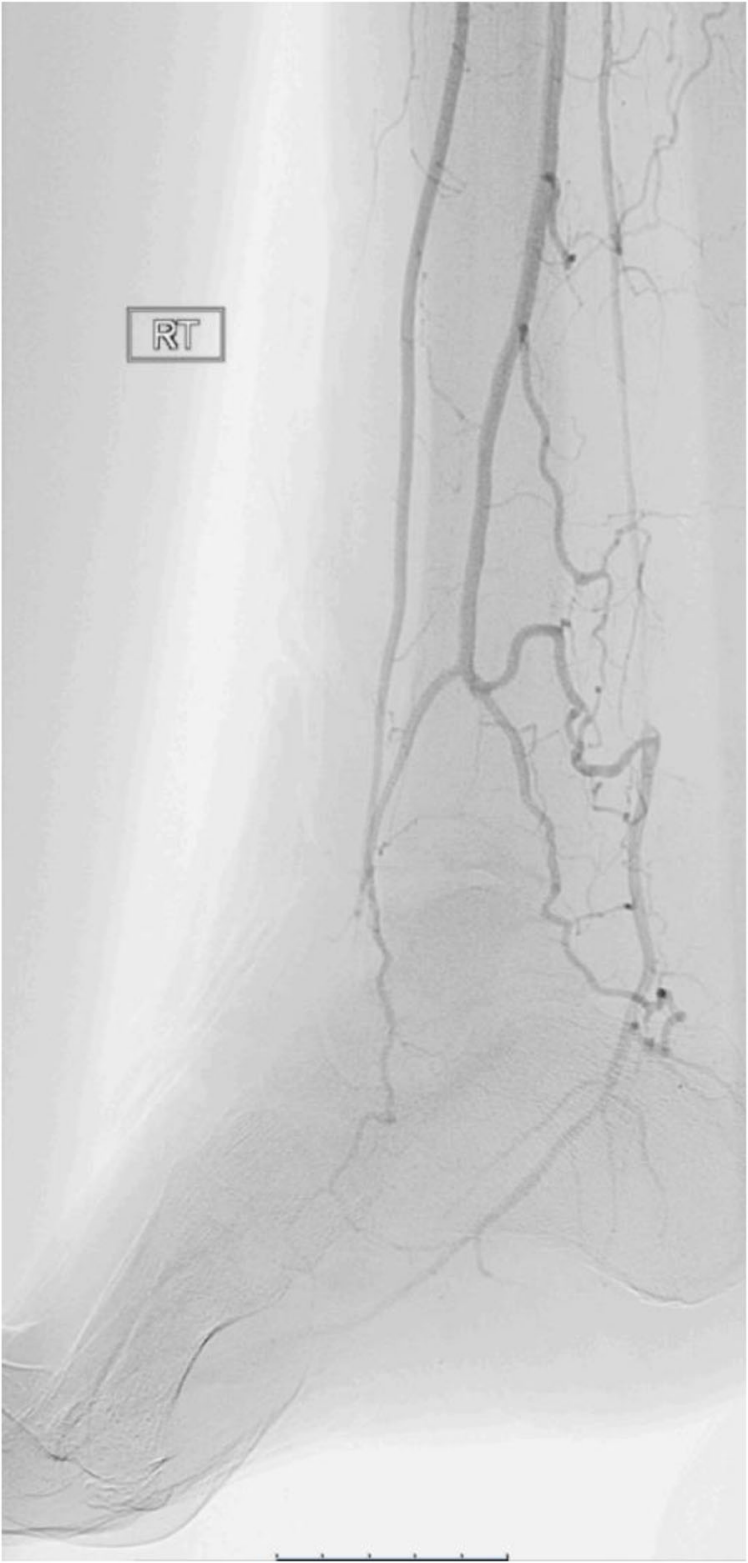
Runoff to the foot after the procedure

**Fig. 9:**
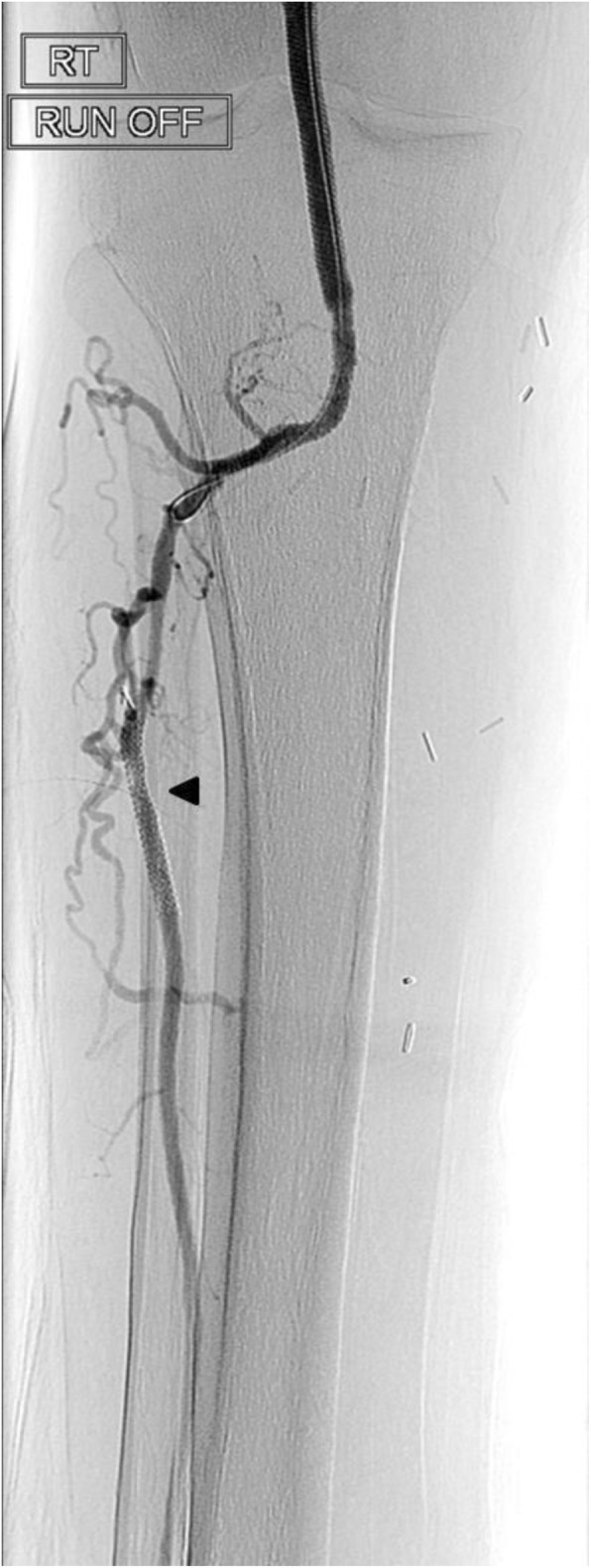
Magnified Fig. 7 at the level of the below-the-knee vessels where the occluded segment of the anterior tibial artery has been recanalized and stented (arrow head) also popliteal artery with a stent

The final angiogram demonstrated brisk inline flow from the R CFA to the origin of the dorsalis pedis artery (Figs. 5, 6, 7 and 8). No significant stenosis or contrast extravasation was noticed at the level of the distal access (Fig. 9).

The Duplex showed patency of all the stents at three, six, twelve, twentyfour months follow up with strong monophasic flow at the DP level (velocity 70 cm/s).

## Discussion/conclusion

Below the knee vessels are low calibre arteries. By inserting the Outback sheath-less, the dimension of the puncture here could be reduced from 8F (outer diameter of the otherwise necessary 6F sheath) to 5.9F, comparable to the outer diameter of a standard 4F sheath used in puncturing crural arteries (JOS CVDB 2016).

This was partly possible by the off-Instructions For Use (IFU) advancement of the device over a more supportive 0.018 wire instead of the recommended 0.014 wire.

A potential issue of advancing the device of an 0.018 wire is abrasion of the hydrophilic coating, release of polymer fragments, separation of the wire, or inability to withdraw the OUTBACK® Elite Re Entry Catheter over the guide wire.

No damage of the vessel was noticed after withdrawing the distal access and no significant stenosis was identified at this level at the subsequent Duplex scans.

The retrograde use of the Outback device, inserted from a tibial vessel in a sheath-less fashion proved to be safe and effective in this case. This, despite having never been described, actually falls under the IFU that just specify that this device should not be used in the coronary and cerebral vasculature; the need to be inserted through a sheath is also never mentioned in the IFU. The risk of arterial injury could be compared to the insertion of a 4F sheath.

The accuracy of the re-entry by this device allowed us to re-enter in short stumps or at the level of the distal CFA without affecting the deep femoral artery.

The outcome of more cases will be decisive for the broader applicability of this technique.

## Data Availability

Data sharing is not applicable to this article as no datasets were generated or analysed during the current study.

## Abbreviations

ATA: Anterior tibial artery
CFA: Common femoral artery
POP: Popliteal artery
SFA: Superficial femoral artery
TPT: Tibioperoneal trunk
